# Candid Remarks on Mr. John Bell's Principles of Surgery

**Published:** 1803-08-01

**Authors:** 


### Candid Remarks on Mr. John Bell's Principles of Surgery: by an Old Member of the London College of Surgeons.

### I. *On the compression of large blood vessels in surgical operations.*

An opportunity has recently occurred to me of perusing several pages of a splendid and expensive work,* entitled, "Principles of Surgery," the first volume of which is "presented by the Author to the Surgeons of London." So magnificent and valuable a present must be received by them as an indubitable proof of the author's discernment and esteem; it will not only be considered as a fresh mark of his anxiety for the public welfare, but as an evidence of his affectionate concern for the reputation and professional skill of the London Surgeons. This being the *third systematic work*† of the kind, which has lately arrived from the metropolis of North Britain, practitioners in London will, doubtless, be ever ready to acknowledge their obligations to the surgeons of Edinburgh, for thus zealously stepping forward in aid of their improvement.

I am myself a genuine disciple of the London school; and indeed, it has never been my good fortune to visit the famous universities of Scotland, although if I were to judge by their voluminous productions, the northern surgeons have a much greater facility of teaching and writing than my tutors in the south. Some of the lessons which I have learnt from the Hunters, the Bromfeilds, the Potts, the Clines, and the Blizards of London, are so entirely different from

---

* The first volume, *i. e.* all that has yet been published, is sold for no more than Four Guineas, in boards!

† The two former Systems of Surgery were by Mr. Benjamin Bell, and Mr. Latta, of Edinburgh.

from those of our Scottish masters, that I feel it a difficult task to decide who is in the right; but, being a little prejudiced in favour of my own school, I almost am jealous lest any of the London students should be misled by the high authority of the BELLS.

The prejudice in favour of my old tutors has not so far prevailed, as to close my eyes against improvements arising from any other quarter; it does, however, so far predominate, that I cannot patiently bear to see respectable surgeons of London treated as men without common sense, or chirurgical knowledge! This abusive and acrimonious spirit is much more conspicuous in the writings of Mr. JOHN BELL, than in those of his temperate namesakes, whom he therefore stigmatises as dull "phlegmatic" beings.* But it forms no part of my present plan to notice the illiberal reflections and personalities of Mr. JOHN BELL, nor to point out *the minor inaccuracies* which appear in his writings; "SUCH ERRORS, HOWEVER DANGEROUS TO THE AUTHOR'S REPUTATION, WILL NOT MISLEAD THE YOUNG SURGEON, FOR WHOSE USE THIS SYSTEM IS DESIGNED."†

A few weeks ago, on observing a quotation in the Medical and Physical Journal, for June 1803, p. 501, I was directed to that part of Mr. BELL's volume which describes one of the modes of restraining hæmorrhages; Mr. BORRETT has therein cited the opinion of Mr. Bell, with all that deference which is due to his celebrated name, and seems modestly afraid lest the successful event of his own practice should encourage other surgeons to follow his example in similar cases. Mr. Borrett, it appears, was educated in one of the London schools; he adopted the principles of Mr. CLINE and ASTLEY COOPER, which, in my humble opinion, under such circumstances, were highly commendable, and deserving of imitation. The case brought forward by this young surgeon is of the utmost importance and utility, as it tends to establish a practice which Mr. JOHN BELL has endeavoured to discourage. Mr. Borrett has "had the most satisfactory demonstration, that the artery (of the thigh) may be effectually compressed at the groin." Mr. CLINE, Mr. LUCAS, and Mr. COOPER, had also received satisfactory proofs of the same fact; all which proofs are detailed in the Medical

* Vide Dawplucker's Remarks on the first volume of Mr. Benjamin Bell's System of Surgery, p. 11, &c.

† See the author's Advertisement to Vol. I. of his Principles of Surgery.

dical and Physical Journal. Mr. Borrett, however, seems to have regarded this mode of restraining an hæmorrhage, in the lower extremity, as a *novel* experiment, which was first taught him in the year 1795, by Mr. A. Cooper; he was therefore very properly searching for further evidence in confirmation of that practice, not knowing that it is extremely common on the Continent, and has been often had recourse to in the London hospitals.

The opinion which Mr. Bell has advanced respecting the compression of the crural and brachial arteries at their origin, are likely to paralyse the efforts of young practitioners, at a moment when the greatest boldness and promptitude of action are required. This question, therefore, becomes a matter of general concern, and merits the fullest investigation. But, to prevent misconception and unfairness, it will first be proper to quote Mr. Bell's own words on this subject.

"On the difficulty of compressing the great Arteries at the Axilla or Groin," p. 414, &c.

"In performing our operations on the great arteries of the arm pit or groin, we must trust nothing to assistants, nothing to compression of the artery; we must do our operation with peculiar boldness and skill, otherwise we shall hardly save our patient, for in a single moment he is either safe or dead.

"Camper was the first who imagined that the axillary artery might be compressed above the place where the tourniquet can be applied; he demonstrated the fact; he put the pipe of a syringe into the right subclavian artery; he threw water along the arteries, and allowed it to flow out, by opening the left axillary artery; he then applied his thumb upon that artery, *below* the clavicle, and found, that by a moderate pressure he could resist the stroke of the syringe, and prevent the water from flowing out. Mr. Blizzard was the next who spoke of this observation, as of importance, especially to the military surgeon. Perhaps, there could not be a more patriotic and sensible thing, than to give a lesson on the application and use of the tourniquet to the cadets of the artillery school; and in a discourse on this subject, we find Mr. Blizzard giving a slight sketch, by Cipriani, of the manner of applying the compress, and of fixing it by pressing with the thumb *above* the clavicle. The expectation excited by so natural a proposal, so well supported by anatomy and experiment, if not by experience, was very great. It was very natural to suppose suppose that it must be equally easy to compress the femoral artery at the groin: at last it came to be universally believed, that the surgeon had no reason to be afraid; that if he failed to suppress any hæmorrhages, it must be for want of skill; that the fixing of the thumb over the clavicle, or groin, was as effectual as the application of the tourniquet itself. What good, or harm, may have been done by this invention it were presumption in me to say; but this much it is my duty to mention to you, that this method of compression is *insecure*; that it will be apt to fail you in your greatest need; that the living circulation, and the struggling of a man under the tortures of an operation, are very different from the experiment by Camper with an anatomical syringe on a dead body.

"I will also tell you what I have actually seen. In an aneurism of the axilla, the surgeon trusting to the compression which his assistant made above the clavicle, opened the tumor, was deluged with blood, and saved the patient only by striking a great needle under the artery! he saved his patient for the moment, but could not save his life; he died of hæmorrhagy in a few days.

"A friend of mine appointed myself and another surgeon to compress the artery of the groin, while he performed the operation for femoral aneurism. Our cushion was well made; our pressure upon the artery, in making the experiment before hand, stopped at once the pulse in the foot, and the throbbing of the aneurism; we pressed much more firmly during the operation; I fixed the compress with all my force; the other gentleman lay over me, and supported my thumbs with his. We had every reason to believe that we had a perfect command of the artery; yet at the very first stroke of the knife, the surgeon having cut into the aneurism, was covered with blood, and saved his patient with difficulty.

"Mr. Allanson was called to a man who had been blown out at the port-hole of a ship by his gun going off while he was ramming home the charge, and found the arm so shattered, that he was forced to amputate at the shoulder joint. In performing this operation he found that the compression of the subclavian artery, even in a man who was lying quite stupid from the violence of the explosion, had not the slightest effect upon the artery; for having begun his incision on the part the most distant from the axilla, on the outside of the arm, he found the smallest branches bleeding profusely, and his expression is remarkable:— '*after which an arterial branch discharged so freely that*

*we*

*we were convinced the pressure on the subclavian artery was not effectually though judiciously made.* (Allanson,' p. 185.)

"Conclusion.—I will repeat with confidence what I have formerly affirmed, *that it is one thing to suppress the pulse in the lower part of the limb, and another thing to stop the pulse in the great artery.*—I have tried in great operations, near the trunk of the body, to stop the blood by pressure; but though I could suppress the pulse of the femoral artery with my fore finger, I could not command its blood with the whole strength of my body.*

"I beseech you then, do not deceive yourselves with any such expectation; do not believe that the effect of this pressure is such as to allow you to perform a quiet, deliberate operation; look upon this operation as one where you have to deal with an open artery, and lay your account with being covered with blood the moment you open the sac. Nor must you ever expect to clear the great cavity of blood, so as to enable you to see the bleeding artery; it will, on the contrary,

---

*"The fact which I have here affirmed, is of too much importance for me not to maintain it with more than common earnestness. I affirm then, that—though the throbbing of an aneurism, or the pulse in the lower part of a limb be quite suppressed, yet the circulation is not stopped, and I entreat the young surgeon never to trust to any such mark of the compression being effectual. This I have formerly affirmed in my Anatomy of the Arteries, and will continue to affirm (as Luther said) though as many devils as there are tiles in Bath were combined against me. It is because I have affirmed this, and because I have ventured to doubt, that compressing the carotid Arteries will cause sleep, cure convulsions, and restore maniacs! that Dr. Parry, of Bath, has run out upon me the whole length of his chain; he has abused me in a magazine, and, if I am rightly informed, has written lately a book on Angina Pectoris, where I am not spared:: I have had retention enough not to answer Dr. Parry in his magazine; yet he is not to suppose that I will let myself be worried peaceably.

" Dr. Parry asks, Whether the ceasing of the pulse in the lower part of the limb be not a certain mark of the circulation being suppressed? And, whether there be any other mark by which the Surgeon can know when his tourniquet is effectually applied? I am confounded at such questions coming from a gentleman of Dr. Parry's rank in his profession. The surgeon always screws the tourniquet till he suppresses the pulse in the lower part of the limb; but he never knows whether he has suppressed the circulation, whether the artery be effectually compressed till he has begun his operation, and then if the arteries of the limb bleed, he screws this tourniquet a little firmer. Once, while I was performing the operation for aneurism in the arm, although the tourniquet was so applied as to suppress both the pulse of the wrist and that of the aneurism, the instant the incision was made the blood flowed in full stream. My assistant screwed, and screwed again; he actually undid the tourniquet in the middle of my operation; took it away, and applied another, and then I finished my operation. This happened in a public hospital.

"Am I a dog that you are come out against me to beat me with staves." *Bell's Principles, p.* 416.

trary, fill the cavity with blood faster than you can bale it out, till the patient breathes his last. Operate in such cases with that rapidity and decision which can alone ensure your patient's safety: there are occasions, in which the old seducing word *Caution* is full of danger."

At page 426, Mr. Bell, among his RULES OF PRACTICE, says, "When the tourniquet cannot be applied, trust nothing to the compression at the axilla, or groin; for though it is sometimes effectual, it often fails, and lives have been endangered by a surgeon's entering on such operations without being prepared for the worst. You find by making the experiment on your own arm, that you can stop the pulse; you think yourself able to secure the patient; but it is, I protest, a mere deception, for the circulation is not suppressed when the pulse ceases, or the pulsation of the tumor prevented."

In the 2d. volume of his Anatomy, 8vo. Edinburgh, 1797, p. 359, are these words, "The old story of compressing the axillary artery above the clavicle, is now of no credit with any surgeon of knowledge and good sense;" and at p, 257, Mr, Bell tells us, "After having employed all his strength in attempting to restrain the bleeding of the femoral artery, he has seen it, with horror, rush as freely as if he had not been there."

These then, are Mr. Bell's opinions, and facts. I shall now subjoin some observations and evidence, which induce me to believe there are men "*of knowledge and good sense,*" on the other side, whose sentiments deserve attention.

*London, July* 20, 1803.

[ To be continued. ]

